# Long-Term Follow-Up after Conservative Surgical Treatment of Odontogenic Myxoma: A Case Report and Literature Review

**DOI:** 10.1155/2019/1634842

**Published:** 2019-02-11

**Authors:** Hitoshi Sato, Yuji Kurihara, Sunao Shiogama, Kotaro Saka, Yuya Kurasawa, Masakatsu Itose, Atsutoshi Yaso, Tatsuo Shirota

**Affiliations:** Department of Oral and Maxillofacial Surgery, School of Dentistry, Showa University, 2-1-1 Kitasenzoku, Ota-ku, Tokyo 145-8515, Japan

## Abstract

Odontogenic myxoma (OM) is a relatively rare, benign odontogenic tumor with locally aggressive behavior, but it is a nonmetastasizing neoplasm of the jaw bones. Although radical resection with an appropriate surgical margin is recommended, emerging evidence has suggested that a more conservative approach will result in acceptable recurrence rates with less morbidity if careful long-term follow-up is provided. A 56-year-old Japanese man with odontogenic myxoma of the left mandible was conservatively treated by surgical enucleation and curettage because he desired functional and cosmetic preservation. During a follow-up period of 100 months, the patient has remained clinically and radiologically free of recurrence. As far as we can ascertain, 20 reports published after 1990 described 37 patients with mandibular OM that had been treated by conservative surgery. Tumors recurred during a mean follow-up of 49.2 ± 42.8 months in 7 (18.9%) patients, and only one of five patients who were followed up for over 100 months developed recurrence. The rate of recurrence decreased from 24.0% to 8.3% when follow-up exceeded 60 months. Although enucleation and curettage have proven effective, the risk of recurrence remains considerable and long-term follow-up is indispensable. More evidence of long-term outcomes after conservative surgery for OM is needed.

## 1. Introduction

Odontogenic myxoma (OM) is a benign, locally invasive, aggressive, nonmetastasizing neoplasm of the jaw bones. It is the third most common odontogenic lesion with an annual incidence of ~0.07 new patients per million people [1, 2]. Although OM is a benign tumor, radical treatment is needed because the rate of local recurrence ranges from 10% - 33% [3-5]. Surgical resection with a minimum bone margin of 1 cm has been advocated [6-9], but emerging evidence suggests that a more conservative surgical approach will result in acceptable recurrence rates with less patient morbidity if follow-up can be over the long term [7, 10-12]. However, little has been reported about the actual long-term follow-up of patients with OM after conservative surgery. Here, we describe a mandibular odontogenic myxoma that was treated by conservative surgery and followed up for 100 months thereafter.

## 2. Case Report

### 2.1. Patient Information

**Figure 1:**
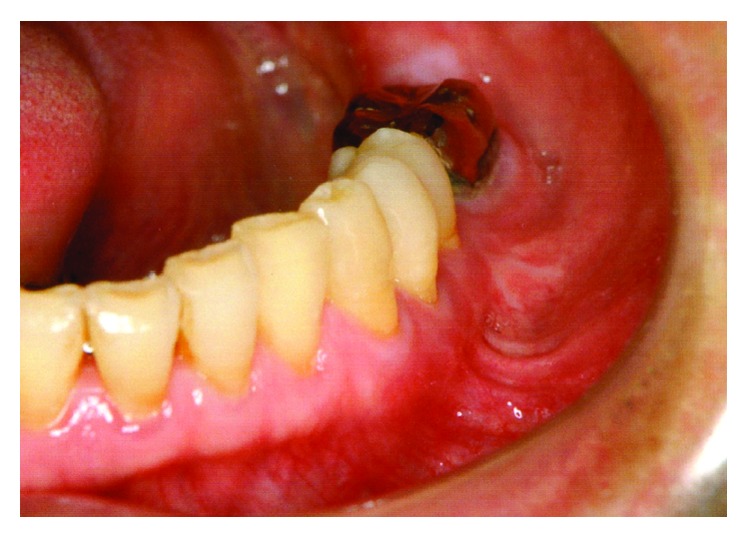
Intraoral photography before surgery. The image shows no symptoms such as redness or swelling of mucosa in the mandible.

**Figure 2:**
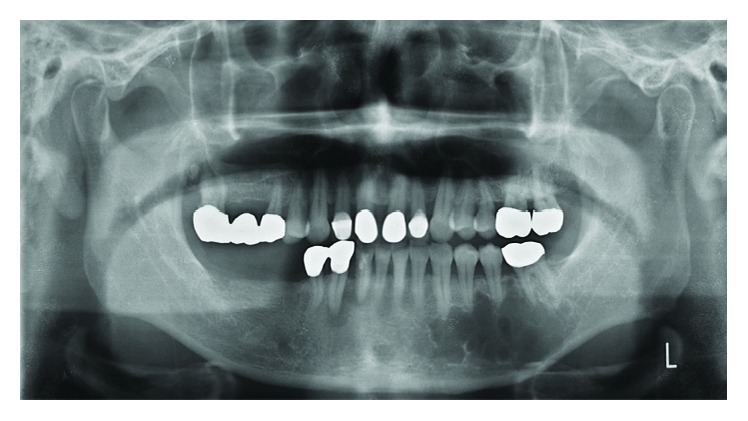
Panoramic dental radiography before surgery. The image shows extensive multilocular radiolucent area with imprecise borders and “soap bubble appearance”.

**Figure 3:**
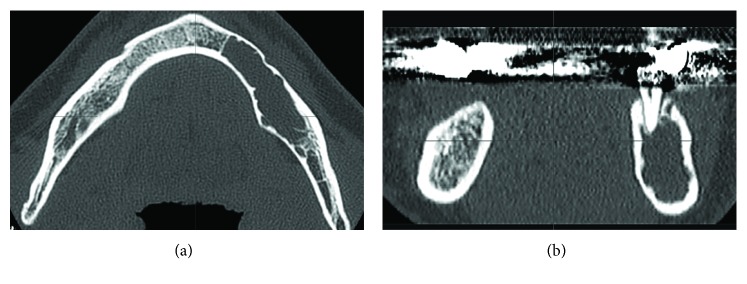
Computed tomography (CT) image before surgery. The axial (a) and coronal (b) CT images show tumor infiltration of cortical bone extending to the inferior mandibular border.

**Figure 4:**
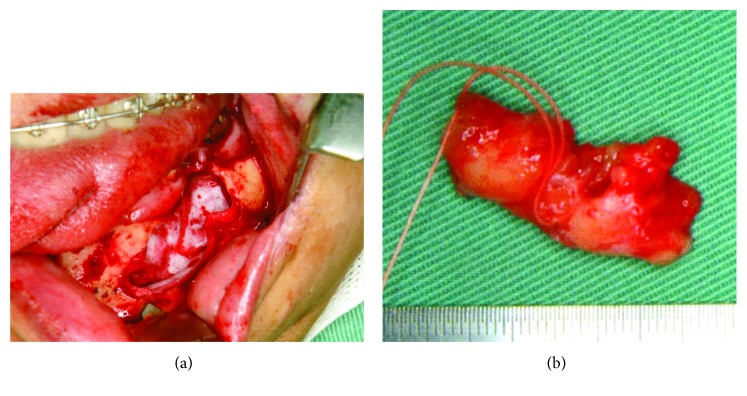
Surgical procedures and resected specimen. The total enucleation and wide curettage of the surrounding bone (a) and resected specimen (b).

**Figure 5:**
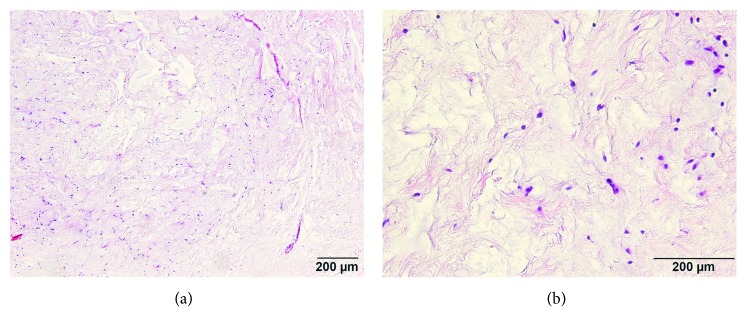
Pathophysiological findings of stained specimen. Hematoxylin and eosin stain ×100 (a) and ×400 (b) magnification.

**Figure 6:**
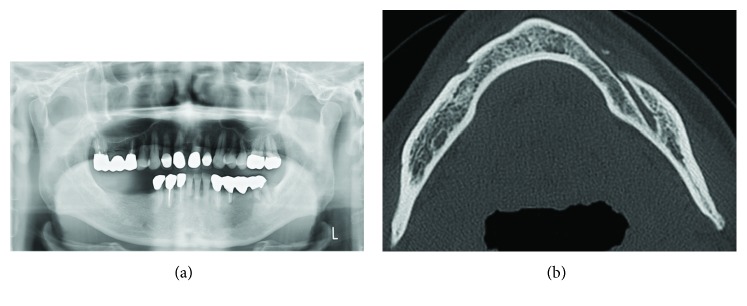
Images of the jaw at 96 months after surgery. The panoramic dental radiograph (a) and computed tomography image (b) show no signs of recurrence.

A 56-year-old Japanese man was referred from a dental clinic for further examination of a radiolucent finding on the left side of his mandible in January 2010. The patient reported having no symptoms in his mouth including the left mandible area. His medical and dental history was noncontributory. An extraoral examination upon initial presentation revealed unremarkable findings and no complaints of paresthesia. An intraoral examination also confirmed the absence of redness and tender swelling of the left mandibular mucosa (Figure 1). Panoramic radiography revealed an extensive multilocular radiolucent area with imprecise borders and a “soap bubble appearance” (Figure 2). Computed tomography showed an approximately 39 × 19 × 11 mm tumor that extended to the roots of four teeth (#33 - 36; Figures 3(a) and 3(b)). We considered that the odontogenic tumor was benign and an incisional biopsy was performed under local anesthesia. The histopathological findings revealed loosely arranged spindle-shaped cells in a myxoid fibrous stroma, indicating a clinical diagnosis of an odontogenic tumor. Segmental resection of the mandible was planned. The patient was given repeated and careful explanations about the high likelihood of recurrence, but he insisted upon a more conservative approach as he desired functional and cosmetic preservation. Conservative surgery then proceeded under general anesthesia after endodontic treatment of #33 – 36 was completed. The surgery consisted of extracting the second premolar from the left mandible, followed by total enucleation and vigorous curettage of the bone (Figure 4(a)). The surgical specimen (Figure 4(b)) revealed apparently benign, spindled-shaped cells in a loose and abundant mucoid stroma (Figures 5(a) and 5(b)). These findings confirmed the diagnosis of odontogenic myxoma. The immediate postoperative period and wound healing were uneventful. The patient underwent monthly clinical examinations for the first year thereafter, then every two months during the second year. Panoramic X-rays were obtained every three months for the first two years. Annual follow-up for eight years included panoramic X-rays and CT imaging (Figures 6(a) and 6(b), respectively), which showed no clinical or radiological signs of recurrence.

## 3. Discussion

Odontogenic myxomas are very rare (<10% all odontogenic tumors) benign tumors of the ectomesenchymal and/or mesenchymal origin [13, 14]. They are locally invasive and slow-growing, and their pathological characteristics in the tooth-bearing areas of the mandible and maxilla are well defined [13-17]. The radiographic features are described as those of a multilocular lesion with a “soap bubble” or “spider web” appearance [14-17]. Such lesions are often discovered incidentally during routine dental checkups, and about 60% of patients are in their second or third decade of life [4, 18]. Although the present patient was asymptomatic, panoramic radiography of the left mandible revealed an extensive radiolucent and multilocular area with imprecise borders that extended from the root of tooth #33 to that of tooth #36.

The treatment strategy for OM remains controversial. Because OM is locally aggressive and it can potentially cause extensive bony destruction, the treatment of choice seems to be radical surgery such as segmental resection. Indeed, complete surgical removal with curettage and peripheral ostectomy alone seems insufficient since OM is not encapsulated and myxomatous tissue infiltrates adjacent bone [3, 6-9]. Thus, the only initial treatment option for extended OM in principle is surgical resection because of the high risk of recurrence which reportedly ranges from 10% to 33% [4, 5]. In addition, recurrence rates can reach 25% after simple enucleation and curettage alone [19, 20]. On the other hand, some reports described that the choice of current recommended therapy depends on the size of the lesion and its nature and behavior between curettage and radical excision [3, 7]. In the present case, CT had shown that the tumor included the #33 - #36 apex and extended to the inferior border of the mandible. Therefore, radical resection such as block resection for mandible was strongly recommended. However, the patient rejected this strategy because he desired functional and cosmetic preservation, and in fact, conservative treatment with enucleation, curettage, and marginal resection would confer several advantages over the radical approach. It is substantially less invasive, it can be surgically implemented via an intraoral approach, and it offers the possibility of preserved nerve function and aesthetics and a shorter stay in the hospital. Recent evidence suggests that a more conservative approach will result in acceptable recurrence rates with less morbidity to patients if long-term follow-up is provided [7, 10-12].  Table 1 describes 20 reports of 37 patients with that was treated by conservative surgery [4, 6, 7, 10, 11, 17, 18, 21-33]. The mean age of the patients was 27.0 ± 15.2, and 20 (54%) were female. The tumors in almost all of them were managed by enucleation and curettage and recurred in 7 (18.9%). One and six of the procedures with recurrence had undergone marginal resection and enucleation with curettage, respectively. The patients were followed up for a mean of 49.2 ± 42.9 (range, 2 – 180) months. Including the present patient, only five have been followed up for over 100 months and the tumor recurred in one of them. The recurrence rate among all patients who were treated by conservative surgery was 19.0%. This rate is relatively lower than that previously described [19, 20]. Furthermore, our investigation of the literature indicated that the rate of recurrence decreases from 24.0% to 8.3% when the follow-up period exceeds 60 months.

The main reason for recurrence is thought to be incomplete removal rather than the intrinsic biological behavior of the OM [34]. Although the tendency is towards a more conservative surgical approach for children and a more radical approach for adults, Kansy et al. does not support this management strategy because the recurrence rates between enucleation and segmental resection are similar [17]. Tumor size has recently been considered to be a factor in the choice of a radical or more conservative surgical approach [4, 10, 11]. Conservative surgical procedures such as enucleation and curettage are recommended when the diameter of OM is <3 cm, whereas a radical approach such as segmental resection with immediate reconstruction is preferred when patients have larger tumors [11]. The conservative surgical recommendation is to enucleate a lesion with wide curettage of normal tissue or a generous amount of apparently intact tissue or even marginal resection of the mandible [12]. This approach has the advantage of preserving vital structures and maintaining oral function, and it could be applied to treat OM that recur after simple surgery [12]. However, one patient who developed recurrent OM 15 years after the initial procedure including tumor resection with the preservation of mandibular continuity has been described [17]. More radical surgery is inevitable for a large number of patients due to a tendency towards more extensive OM with significant destruction of key structures [17, 27]. Moreover, the rate of OM recurrence remains vague because few reports described long-term follow-up after conservative surgery. Thus, more evidence about long-term outcomes after conservative surgical treatment of OM is needed.

## 4. Conclusions

A conservative surgical approach comprising enucleation and curettage can be effective for OM management. Recurrence rates decreased from 24.0% to 8.3% among patients who were treated with conservative surgery and followed up for over 60 months. The risk of recurrence is likely to be considerable, and long-term follow-up is indispensable for the conservative management of OM.

## Figures and Tables

**Table 1:** Clinical reports of mandibular odontogenic myxoma published after 1990.

No.	Author	Year	Age	Sex	Follow-up period (months)	Treatment	Recurrence	Size (mm)
1	Oliveira et al. [21]	2018	9	F	6	E/C	None	NA
2	Albanese et al. [22]	2012	25	F	6	E	None	21.2 × 47.6
3	Mauro et al. [23]	2012	6	M	6	E/C	None	NA
4	Subramaniam et al. [7]	2016	16	—	7	M	None	NA
5	Shivashankara et al. [24]	2017	13	M	12	E/N	None	40 × 20
6	Subramaniam et al. [7]	2016	18	—	12	E	None	NA
7	Miranda Rius et al. [25]	2013	55	M	12	E/C	None	33 × 28
8	Hammad et al. [26]	2016	45	F	13	M	None	50 × 30
9	Francisco et al. [27]	2017	9	F	14	E/C	Recurrence	NA
10	Francisco et al. [27]	2017	12	F	16	E/C	Recurrence	NA
11	Sumi et al. [28]	2000	48	M	22	E/C	None	70 × 25 × 15
12	Mittal et al. [29]	2016	48	F	24	E/C	Recurrence	25 × 20
13	Lin and Basile [30]	2010	25	F	24	E	None	NA
14	Lo Muzio et al. [4]	1996	21	M	24	E/C	None	NA
15	Lo Muzio et al. [4]	1996	28	M	24	E/C	Recurrence	NA
16	Bucci et al. [31]	1993	28	M	24	E/C	None	43 × 40
17	Francisco et al. [27]	2017	7	F	26	E/C	None	NA
18	Francisco et al. [27]	2017	15	F	27	E/C	Recurrence	NA
19	Lo Muzio et al. [4]	1996	16	F	31	E/C	None	NA
20	Francisco et al. [27]	2017	30	F	34	E/C	None	NA
21	Boffano et al. [11]	2011	38	M	38	E/C	None	25
22	Boffano et al. [11]	2011	42	F	40	E/C	None	30
23	Boffano et al. [11]	2011	20	M	42	E/C	None	20
24	Rajasekhar et al. [32]	2008	17	F	48	M	None	70 × 30
25	Lo Muzio et al. [4]	1996	22	F	48	E/C	Recurrence	NA
26	Takahashi et al. [6]	2018	37	F	73	E/C	None	40 × 19 × 12
27	Chaudhary et al. [33]	2015	25	F	84	E/C/M	None	NA
28	Li et al. [18]	2006	7	M	84	E/C	None	NA
29	Li et al. [18]	2006	32	M	84	E/C	None	NA
30	Lo Muzio et al. [4]	1996	65	F	84	E/C	None	NA
31	Francisco et al. [27]	2017	17	M	85	E/C	None	NA
32	Francisco et al. [27]	2017	11	F	98	E/C	None	NA
33	Present case		56	M	100	E/C	None	39 × 19 × 11
34	Francisco et al. [27]	2017	27	F	117	E/C	None	NA
35	Kawase-Koga et al. [10]	2014	40	M	120	E/C	None	40 × 30 × 15
36	Li et al. [18]	2006	37	M	132	C	None	NA
37	Kansy et al. [17]	2012	33	F	180	M	Recurrence	NA

Abbreviations: M: male, F: female, E: enucleation, C: curettage, M: marginal resection, and NA: not available.
